# Discriminant Analysis of Raman Spectra for Body Fluid Identification for Forensic Purposes

**DOI:** 10.3390/s100402869

**Published:** 2010-03-29

**Authors:** Vitali Sikirzhytski, Kelly Virkler, Igor K. Lednev

**Affiliations:** Department of Chemistry, University at Albany, SUNY, 1400 Washington Avenue, Albany, NY 12222, USA

**Keywords:** body fluids, semen, blood, saliva, forensic medicine, human, discriminant analysis, spectroscopy, spectrum analysis, Raman spectroscopy

## Abstract

Detection and identification of blood, semen and saliva stains, the most common body fluids encountered at a crime scene, are very important aspects of forensic science today. This study targets the development of a nondestructive, confirmatory method for body fluid identification based on Raman spectroscopy coupled with advanced statistical analysis. Dry traces of blood, semen and saliva obtained from multiple donors were probed using a confocal Raman microscope with a 785-nm excitation wavelength under controlled laboratory conditions. Results demonstrated the capability of Raman spectroscopy to identify an unknown substance to be semen, blood or saliva with high confidence.

## 1. Introduction

The body fluid traces recovered at crime scenes are among the most important types of evidence to forensic investigators [1]. Conventional methods of body fluid identification use labor-intensive, technologically diverse methods that are performed one after the other and are costly in terms of time and sample usage [2,3]. The objectives of any crime scene investigation are to preserve physical evidence and collect only valuable evidence for the analytical examination [3]. So, the ability to characterize an unknown stain at the scene of the crime without destruction and having to wait for laboratory results is a very critical step in crime scene investigation.

Although several methods have been developed over the years for body fluid identification, nondestructive tests that can be performed at a crime scene are still in the developmental stage. Fluorescence and Raman spectroscopies are among the most promising nondestructive methods for confirmatory identification of body fluids [4–12]. The less sensitive Raman spectroscopy in comparison with fluorescence has higher selectivity and specificity to biochemical species and could potentially be useful in resolving mixtures of multiple body fluids with minimal sample preparation or manipulation [4–11,13]. We have recently demonstrated that the combination of Raman spectroscopy and advanced statistics can potentially discriminate human and animal blood traces [13,14]. Raman scattering is a powerful qualitative and quantitative analytical method based on a process where incident monochromatic photons interact with a sample to produce scattered photons with an energy distribution characteristic of molecular structure [15–17]. In the modern era of digital light detectors providing hundreds and thousands of data points in every measured spectrum, statistical analysis becomes an especially powerful tool. The once golden rule of previous generations of spectroscopists–that if you do not see a change in the spectrum by naked eye, then you are chasing a ghost–no longer applies [18]. Advanced statistical methods allow for retrieving of reliable information from data sets that is not otherwise evident. We have been successful in developing and applying advanced statistical analysis of Raman spectra for biochemical [19–27] and forensic purposes [7–9,11,13].

For the last four years, our laboratory has been working on the development of a novel approach for body fluid identification based on near Infrared (IR) Raman spectroscopy and advanced statistical analysis. Our approach is based on the hypothesis that the biochemical composition of each body fluid is unique and Raman spectroscopy can easily recognize the difference [10]. We utilize Raman spectroscopy to characterize the entire composition of the fluid instead of probing a specific chemical group or compound. Each body fluid has a complex biochemical composition and becomes heterogeneous when dry trace forms. As a result, Raman spectra acquired at different spots of the same dry trace sample are different, and no single characteristic spectrum can satisfactorily represent the experimental. In addition, one can expect that the composition of body fluids varies from donor to donor. We have recently investigated the effect of dry body fluid heterogeneity and spectral variations due to multiple donors [7–9]. As a result, multi-dimensional Raman spectroscopic signatures have been developed for dry traces of blood, semen and saliva based on Principal Component Analysis (PCA) [7–9]. In this article, we briefly overview our approach for developing a multi-dimensional Raman spectroscopic signature for a single body fluid and discuss our new results on the application of advanced statistical analysis of Raman spectroscopic data for identification purposes.

A multi-dimensional Raman spectroscopic signature was built for each body fluid to uncover the sources of spectral variation and to assign spectroscopic features to the chemical species. However, utilizing these signatures for identification purposes was not optimal. As a more efficient alternative method, we utilized Discriminant Analysis (DA) based on Soft Independent Modeling of Class Analogy (SIMCA), Linear Discriminant Analysis (LDA) and Partial Least Squares Discriminant Analysis (PLS-DA) techniques for body fluid identification purposes.

## 2. Multi-dimensional Raman spectroscopic signature for a single body fluid (review of data published recently)

### 2.1. Experimental

Sets of 50 semen, 14 blood and 15 saliva samples were obtained from anonymous donors and volunteers (see 7–9 references for details). A 10-μL drop of each body fluid sample was placed on a circular glass slide designed for use with an automatic mapping stage and allowed for drying completely. Prepared samples were analyzed on a Renishaw inVia confocal Raman spectrometer equipped with the a research-grade Leica microscope, 20x long-range objective, and WiRE 2.0 software using automatic mapping (lower plate of a Nanonics AFM MultiView 1000 system) that scanned a sample area of 75 × 75 μm and measured Raman spectra from 16–36 spots within the area with 6 ten-second accumulations for each spot. Measurements were taken using Quartz II and QuartzSpec software. The obtained spectra were treated with GRAMS/AI 7.01 software to remove any cosmic ray interference and imported into MATLAB 7.4.0 for statistical analysis. The number and possible identities of principal spectral components were determined for semen, blood and saliva using significant factor analysis (SFA) and the alternate least squares (ALS) function. A multi-dimensional spectroscopic signature of a specific body fluid was built from these principal components.

### 2.2. Raman Spectroscopic Signature of Semen

**Figure 1.**
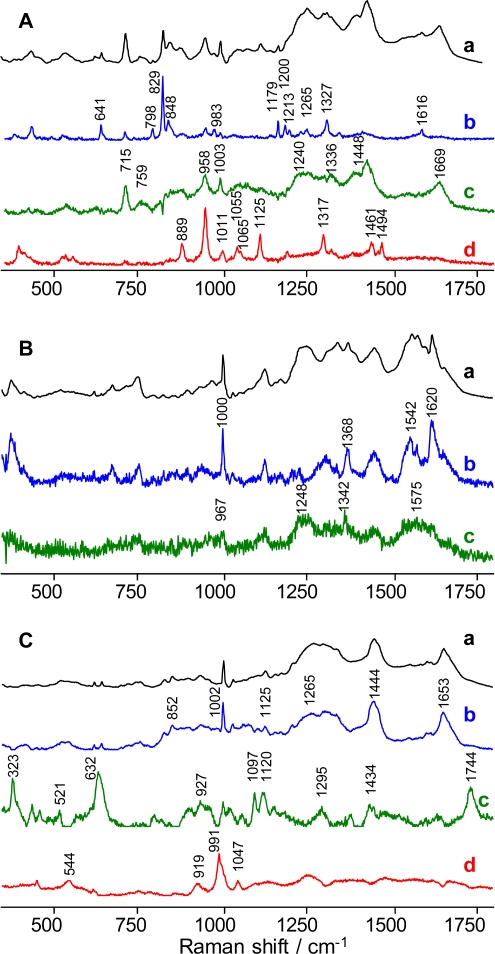
Multi-dimensional spectroscopic signatures of semen (**A**), blood (**B**) and saliva (**C**); average Raman spectra obtained for each body fluid (a, **black lines**), and principal spectral components (b–d; **blue**, **green** and **red** lines).

Alternating least squares (ALS) analysis of the 36 Raman spectra of a single dry semen sample was utilized to build a multi-dimensional spectroscopic signature. It was found that three major principal components satisfactorily represented semen Raman spectra (Figure 1.A). An additional component was used for taking into account a fluorescent background.

Each individual spectral PC was built by contributions from several biochemical components of semen. As is common for ALS, cross-mixing of peaks took place. Peaks which were intense in one PC also appeared in other components, but with lower intensity.

Specifically, the first PC dominated by the contribution from tyrosine [7,28,29] with characteristic peaks at 641, 798, 829, 848, 983, 1179, 1200, 1213, 1265, 1327, and 1616 cm^−1^ showed a noticeable contribution from other biochemical species (Figure 1.A). The second PC is dominated by Raman peaks specific for proteins. In particular, Raman bands at 1668 cm^−1^ and 1240 cm^−1^ known as Amide I [30–32] and Amide III bands [33], respectively, are characteristic vibrational modes of the polypeptide backbone. Peaks at 759, 1003, 1336, and 1448-cm^−1^ closely match the Raman bands of serum albumin [34,35]. The strong peak at 715 cm^−1^ is consistent with the C–N symmetric stretching vibration reported for choline [36–38]. The 888, 958, 1011, 1055, 1065, 1125, 1317, 1461, and 1494-cm^−1^ Raman bands of PC 3 are consistent with previously reported spectra of spermine phosphate hexahydrate (SPH) [39,40]. Component 3 could not be assigned solely to SPH, but it exhibits several peaks also found in the first two principal components.

The three described principal components, combined with a horizontal line and a tilted line presenting the fluorescent contribution, were used to create a multi-dimensional spectroscopic signature of semen. The linear combination of these components fitted any experimental Raman spectrum of all 50 semen samples with high quality [7].

### 2.3. Raman Spectroscopic Signature of Blood

A multi-dimensional spectroscopic signature of dry human blood was developed using the same approach as that for semen. The blood spectroscopic signature which contained three major principal components (Figure 1.B), including a fluorescent background, hemoglobin- and fibrin-dominated spectra, was created based on statistical analysis of Raman spectra recorded from multiple spots of a single dry sample of blood. The component dominated by the fluorescent background was important only for fitting purposes, so the two other components were named as component 1 (hemoglobin-dominated) and component 2 (fibrin-dominated). Component 1 was assigned to hemoglobin and its derivatives due to the presence of peaks at 1000, 1368, 1542 and 1620 cm^−1^(Figure 1.B) [41–43]. The appearance of a hemoglobin-dominated principal component was expected. According to the literature, the dried contents of red blood cells are composed almost completely of hemoglobin and its derivatives [44]. Component 2 was present in a smaller abundance in the dried blood sample. Peaks at 967, 1248 and 1342 cm^−1^ are similar to major peaks of pure fibrin, one of the coagulated blood components [45]. It was found that the liquid and dried blood spectra have obvious differences, which could be explained by the coagulation process when blood dries. Liquid blood is dominated by the hemoglobin principal component, while dried blood has a noticeable contribution from fibrin, which is the protein formed from fibrinogen during coagulation [46].

Hemoglobin- and fibrin-dominated principal components (developed from a single blood sample) together with a horizontal line and a tilted line presenting the fluorescent background were used for fitting the experimental Raman spectra of dry blood samples obtained from 14 donors. A quantitative statistical analysis using sum of squares due to error (SSE), R-square, and root mean squared error (RMSE) was performed to confirm a satisfactory fitting of all experimental spectra [9].

### 2.4. Raman Spectroscopic Signature of Saliva

The process of multi-dimensional spectroscopic signature building was repeated with 15 saliva samples. According to significant factor analysis combined with principal component analysis, near IR Raman spectra of dry saliva samples demonstrated higher variability relative to semen and blood samples. The presence of 11 principal components was detected. Three major components were chosen (Figure 1.C) as a spectral representation of saliva chemical composition. Other components appeared as a fluorescent background, noise and spectra with insignificant contribution to the overall signal. The spectral components of saliva have contribution from multiple chemical species and the assignments are based on the known composition of saliva and literature data. A significant contribution from protein Raman bands is evident in the first spectral component due to the appearance of the Amid I [30–32], aromatic breathing [28] and CH stretching peaks at 1653, 1002 and 1444 cm^−1^, respectively (Figure 1.C). Glycoproteins and mucin could make the major contribution to this component [47,48]. Several strong Raman peaks of the second component are assigned to acetates (632, 1295, 1434, and 1744 cm^−1^) [49–51] and carbohydrates (323 cm^−1^ and 521 cm^−1^) [46,52], which are also present in saliva [53–55]. This spectral component shows (Figure 1.C, curve c) minute spectral regions with a flat horizontal shape (zero intensity level), which are the result of the nonegativity constrain used for calculating meaningful Raman spectral components. The third component contains strong Raman bands at 544, 919, and 991 cm^−1^, which are consistent with the amino acid arginine, but this is a preliminary assignment and more investigation is needed. Despite the fact that the Raman spectrum of dry saliva, in contrast to blood and semen, varies considerably from donor to donor, a linear combination of three principal components, a horizontal line and a line resenting fluorescent contribution constituting a multi-dimensional signature fits to all Raman spectra of dry saliva samples with satisfactory goodness-of-fit statistics.

## 3. Identification of unknown body fluids

Identification of an unknown species based on spectroscopic data is a common statistical problem. Most of these statistical methods can be separated in two main groups, unsupervised (also called exploratory) and supervised methods [56]. Unsupervised methods are used for studying experimental spectral data without a prior knowledge of the object. Hierarchical cluster analysis (HCA), Density-Based Spatial Clustering of Applications with Noise (DBSCAN) or PCA based methods of the dimensionality reduction are more commonly used approaches [57–63].

Supervised methods utilize a prior knowledge about the system by developing classification models based on known spectra [62,64–66]. These methods include Linear DA (LDA), Direct LDA (DLDA), Heteroscedastic LDA (HLDA), Nonparametric DA (NDA), Kernel-based LDA (K-LDA), SIMCA, PLS-DA and Multivariate Analysis of Variance (MANOVA) [67–74]. Each of these algorithms is most efficient for a certain type of data. In cases when the data set characteristics are not known, selection of the algorithm is usually done using trial and error.

As a first step, a variety of statistical approaches were tested to explore the possibility of body fluid identification. It was found that the application of Raman multi-dimensional signatures built by the alternating least squares algorithm for identification purposes decreased the quality of identification relative to the direct usage of conventional DA methods (data is not shown). That is a consequence of different ideologies of these methods. Multi-dimensional Raman signatures were built to uncover the sources of data variation and to assign collected spectral data to real chemical species. In contrast, the DA is based on the orthogonal matrix decomposition, and obtained components or factors explain maximum data variance. The first approach is important for understanding the chemical composition of the system and can be used, for example, to map real species distribution among the tested area. The second approach gives abstract, unique and orthogonal (independent) solutions, which can be used to determine the number of different sources of the variation present in the data and, eventually, allows discrimination. The second approach does not use special constraints, such as non-negativity, unimodality or local-rank, which are necessary for a physically meaningful result.

We report here on the application of SIMCA, PLS-DA and LDA algorithms for the identification of traces of body fluids based on near IR Raman spectroscopic data. The efficiency of each method was tested by various validation methods, such as a “leave one out” or formation of training and test data sets.

### 3.1. Soft Independent Modeling of Class Analogy

**Figure 2.**
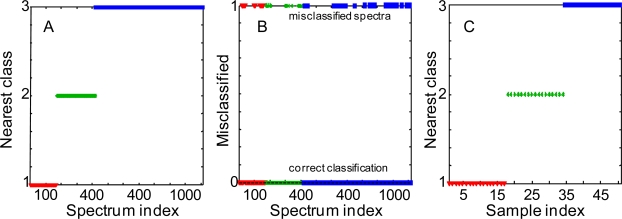
Nearest classes to SIMCA submodels for body fluid samples. Classification of single Raman spectra: correctly classified (**A**) and misclassified (**B**) single Raman spectra. (**C**) Classification of average Raman spectra. Defined classes: human blood (**red triangles**) –class 1, human saliva (**green stars**)–class 2, human semen (**blue squares**)–class 3.

Soft Independent Modeling of Class Analogy (SIMCA) is typically used to identify local models for defined groups and to predict a probable class membership for new observations. SIMCA focuses on modeling the classes rather than finding the optimal classifier. We utilized SIMCA classification method to compare Raman spectra of three body fluids. 170 spectra recorded from 17 blood samples, 252 spectra from 17 saliva samples and 693 spectra recorded from 50 semen samples were used to develop the SIMCA model using three PCA models based on the body fluid types. PCA models were calculated using contiguous block, leave-one-out, Venetian blind, and random subset cross-validation methods for determining the number of latent variables. Hotelling’s T^2^ and Q statistics were used for group membership decisions and to test the normality of principal components obtained from PCA. The results showed that 83% of blood, 88% of saliva and 89% of semen spectra were attributed to the correct models (Figures 2.A, 2.B). These numbers correlate with the total number of spectra reordered for each body fluid, the bigger data set yields the better prediction. To further improve discrimination, all Raman spectra recorded from a particular sample were averaged. Raman spectra of 17 samples of each fluid were treated and used to build a new SIMCA model. This time, 100% of the spectra were correctly classified (Figure 2.C). The success of SIMCA discrimination analysis with PCA data reduction suggests that a special algorithm can be built for the rapid differentiation of body fluids.

### 3.2. Linear Discriminant Analysis

While SIMCA is a very useful classification tool, the PCA submodels in SIMCA are computed with the goal of capturing the variation within each class. Directions in the data space that discriminate classes are not identified in SIMCA. In LDA, linear combinations of variables are computed to determine directions in the spectral space; discriminant functions maximize the variance between groups and minimize the variance within groups according to Fisher’s criterion. For the validation of the LDA model, the leave-one-out cross-validation was used. In this method, all spectra except one were used to build a LDA model and then to classify the left out spectrum. This method is repeated so that each spectrum is predicted once.

**Figure 3.**
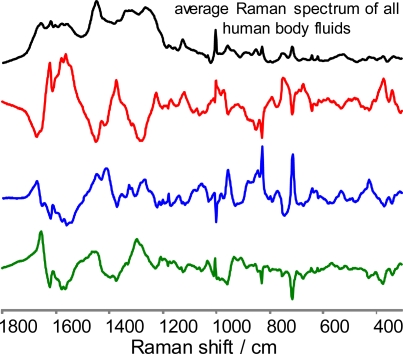
Difference between the average Raman spectrum of all human body fluids (**black**) and averaged Raman spectra of human blood (**red**), semen (**blue**) and saliva (**green**). Spectra are scaled for comparison.

**Figure 4.**
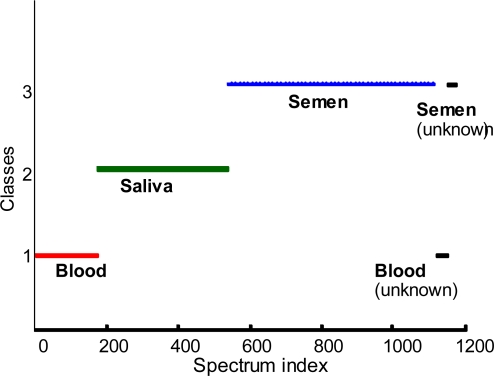
Linear discriminant analysis of the body fluids. Classes defined by training sets: human blood (red)–class 1, human saliva (green)–class 2, human semen (blue)–class 3. Black color indicates unknown spectra.

The difference spectra (Figure 3) demonstrate significant differences between Raman spectra of the body fluids, suggesting a good quality of DA. This conclusion is supported by inspection of the LDA results (Figure 4). In order to decrease the noise contribution and compress the data, all 1115 spectra were treated by multivariate methods such as principal component analysis (PCA) or partial least-squares (PLS) and the resulted score matrix was used for the following LDA as described in the previous paragraph. Each body fluid data was treated separately. PCA and PLS were performed using contiguous block, leave-one-out, Venetian blind, and random subset cross-validation methods. Each colored line (Figure 4) is the superposition of the hundreds of points. Each point corresponds to the specific Raman spectrum of the particular body fluid sample. The position of the point is the result of LDA prediction based on the training set built from the all spectra except the left out spectrum. Red, green and blue colors were designated for human blood, saliva and semen left out Raman spectra respectively. Class 1, class 2 and class 3 correspond to our classification of the training set spectra (Figure 4). False predictions will place colored points in the wrong class. For example, if a human blood Raman spectrum is classified as a Raman spectrum of semen, we should see a red (blood) point in the third (semen) class. Interesting to note, LDA demonstrated 100% discrimination. Assuming that our relatively large set of samples covers a significant part of human body fluid diversity, we can expect a great future using Raman spectroscopy in the field of human body fluid identification. To the best of our knowledge, the demonstrated approach is the only nondestructive method able to determine the species of origin of a body fluid sample. In order to validate this method, we asked a new student, who has not been involved in Raman spectroscopy research projects before, to collect 25 Raman spectra from the four unknown samples. Recorded Raman spectra were treated and discriminated using the described statistical approach. LD analysis of the Raman spectra again demonstrated 100% quality (Figure 4, black line).

Discriminant analysis using naive Bayes classifiers, fitting of multivariate normal densities with covariance estimates stratified by group and Mahalanobis distances also was performed. All these methods can be considered as modifications of LDA. Obtained results were consistent with the previous LDA results. Application of several different methods of discriminant analysis can be important in case of very noisy data or when samples are contaminated.

### 3.3. Partial Least Squares Discriminant Analysis

**Figure 5.**
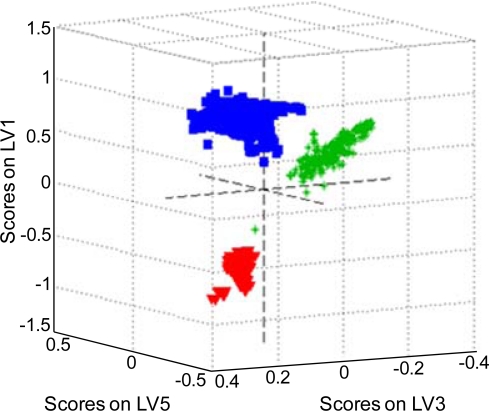
A three-dimensional latent variable plot for human blood (**red**), semen (**blue**) and saliva (**green**) samples based on the first, third and fifth latent variables (LV).

It has been shown that PLS-DA is basically the inverse-least squares approach to LDA, which produces essentially the same result but with the noise reduction and variable selection advantages of PLS [75]. A set of all body fluid Raman spectra was used as PLS-DA input data. For each fluid PLS models, the number of latent variables was calculated using contiguous block, leave-one-out, Venetian blind, and random subset cross-validation methods. Latent variables were used to create three-dimensional plots of the species in space in order to determine if each species would cluster and separate from the other species (Figure 5). Two different sets of latent variables were chosen to show clustering and separation. Figure 5 shows a three-dimensional view based on the first, third and fifth latent variables. All species were separately clustered except for one Raman spectrum of human saliva that fell close to the human blood cluster. The presence of some subclusters in the human blood cluster can be noticed. The sensitivity (number of samples predicted as in the class divided by number actually in the class) and specificity (number of samples predicted as not in the class divided by actual number not in the class) values support visual inspection of the 3D plots (Table 1).

Finally, in order to test the stability of SIMCA, LDA and PLS-DA methods, spectral data with introduced noise and background contributions were analyzed. Such modified data can model real “field” tests when contaminated Raman spectra or spectra with low intensity are recorded. Despite the different resistibility of the tested methods to spectral data quality, obtained results (not shown) allowed us to make a conclusion that the combination of the discriminant methods can be successfully used even for “bad” sets of Raman spectra.

## 4. Conclusions

Detection and identification of traces of body fluids encountered at a crime scene are important aspects of forensic science today. Our previous studies have demonstrated the possibility to characterize body fluids with unique Raman multi-dimensional signatures and assign spectroscopic features to the chemical species. A nondestructive, confirmatory method of body fluid identification using discriminant statistical analysis of Raman data was developed in the reported study. Discriminant Analysis (DA) using SIMCA, LDA and PLS-DA techniques allowed for discriminating semen, blood and saliva trace with 100% probability under laboratory conditions. Several different spectra preprocessing approaches were tested. Averaging Raman spectra acquired for multiple spots on the sample enhanced significantly the discrimination by the SIMCA algorithm. Data reduction by Principal Component Analysis (PCA) and Partial Least Squares (PLS) decomposition was beneficial for DA utilizing SIMCA and LDA family methods. Necessary and sufficient numbers of principal components or latent variables were determined by significant factor analysis. Three-dimensional score plots built for the PLS-DA model (Figure 5) demonstrated clustering among single body fluid samples that could indicate that more specific information about the donor groups is accessible. This investigation is in progress in our laboratory.

Overall, Raman spectroscopy coupled with the discriminant statistical analysis showed great potential for nondestructive, confirmatory identification of body fluids at a crime scene. The ability to make these determinations and identifications, especially on-site at a crime scene, would be a major advance in the forensic analysis of body fluids. Present study deals with pure body fluid traces only. Mixtures of body fluids, contaminations and substrate contributions are important factors for real forensic cases, and our laboratory is working currently on incorporating these additional aspects in the body fluid identification analysis.

## Figures and Tables

**Table 1.** Sensitivity and specificity of PLS-DA. Values calculated for calibration (self-prediction, Cal) and cross-validation results (CV).

Modeled class	Blood	Semen	Saliva
Sensitivity (Cal)	1.000	1.000	0.999
Specificity (Cal)	1.000	0.999	1.000
Sensitivity (CV)	1.000	1.000	0.999
Specificity (CV)	1.000	0.999	1.000
Class. Err (Cal)	0	0.000579	0.000721
Class. Err (CV)	0	0.000579	0.000721
RMSEC	0.0344	0.107	0.103
